# Exosomes: Historical Evolution and Emerging Roles in Dermatology

**DOI:** 10.1111/jocd.16769

**Published:** 2025-01-08

**Authors:** Nina Schur, Luna Samman, Milaan Shah, Victoria Dukharan, Carol Stegura, Luke Broughton, Todd Schlesinger

**Affiliations:** ^1^ Lake Erie College of Osteopathic Medicine Florida USA; ^2^ Department of Dermatology Garnet Health Medical Center Middletown, New York USA; ^3^ Department of Dermatology Medical University of South Carolina Charleston South Carolina USA; ^4^ Department of Dermatology Kansas City University ‐ GME Consortium/Advanced Dermatology and Cosmetic Surgery Orlando, Florida USA; ^5^ School of Medicine, Medical University of South Carolina Charleston South Carolina USA; ^6^ Clinical Research Center of the Carolinas Charleston South Carolina USA

**Keywords:** biomarkers, cell‐free therapy, clinical applications, dermatology, exosomes, extracellular vesicles, nanovesicles, regenerative medicine, skin diseases, therapeutic strategies

## Abstract

**Background:**

Exosomes are a nanoscale extracellular vesicles derived from different cell types that have been investigated for various clinical applications, including functioning as biomarkers and use as direct therapeutics. Given the role of exosomes in multiple pathophysiologic pathways and potential practical applications, they have garnered significant interest in the scientific community but much is still unknown about their development and use.

**Aims:**

This literature review covers the background, mechanisms of action, use as biomarkers, methods of application, and direct therapeutic applications of exosomes.

**Methods:**

A literature review on the background and uses of exosomes was conducted. Key articles describing the pathophysiologic pathways and applications of exosomes were summarized and described.

**Results:**

Exosomes impact several cellular pathways which allow them to function as biomarkers for malignancy and inflammatory dermatoses and may make them useful therapeutics for skin rejuvenation, hair loss, and wound repair. Limitations of exosomes include an incomplete understanding of their functions and impacts and a lack of standardization in their production and application.

**Conclusions:**

Exosomes are a unique and novel cellular medium that offer promise as a diagnostic tool and therapy. While there are limitations to the uses of exosomes as well as our current understanding of them, further investigation may yield additional applications and a larger role in medicine for exosomes.

## 1 Introduction

Exosomes are nanoscale extracellular vesicles that transport cellular materials between cells. Recently, they have garnered attention because of their role in mediating intercellular communication. Ranging from 40 to 200 nm in diameter, exosomes were discovered in the early 1980s and were initially thought to be cellular waste products [1]. However, subsequent research revealed their critical functions in various physiological and pathological processes. Exosomes are recognized as essential carriers of bioactive molecules, including proteins, lipids, and nucleic acids, between cells [2].

Exosomes were first identified during studies on the maturation of reticulocytes, where researchers observed the release of small vesicles containing transferrin receptors. This discovery marked the beginning of a paradigm shift in cell biology, highlighting the potential of exosomes as mediators of intercellular communication [3]. Over the past few decades, extensive research has elucidated the complex biogenesis, diverse composition, and multifaceted functions of exosomes.

Exosomes are distinct from endosomes, although they are intricately linked in their formation process. Endosomes are membrane‐bound compartments within cells that function in the sorting and trafficking of cellular information materials. Early endosomes progressively mature into late endosomes, also known as multivesicular bodies (MVBs), which contain numerous intraluminal vesicles. When these MVBs fuse with the plasma membrane, the intraluminal vesicles they contain are released into the extracellular space as exosomes [4]. Thus, exosomes play a specialized role in extracellular communication through the external trafficking of previously intracellular materials.

Exosomes are involved in several biological processes, including immune responses, cell proliferation, and tissue repair. Furthermore, their involvement in disease mechanisms has been increasingly recognized, with implications for cancer, neurodegenerative disorders, and cardiovascular diseases [4]. The capacity of exosomes to transfer molecular information between cells has opened new avenues for understanding disease pathogenesis and exploring innovative therapeutic strategies. Thus, this literature review aims to provide a comprehensive overview of the background, mechanisms of action, and current and potential diagnostic and therapeutic roles of exosomes. There were no patients involved in this manuscript. The authors confirm that the ethical policies of the journal, as noted on the journal's author guidelines page, have been adhered to. No ethical approval was required as this is a review article with no original research data.

### 1.1 General Properties and Mechanisms of Action

**FIGURE 1:**
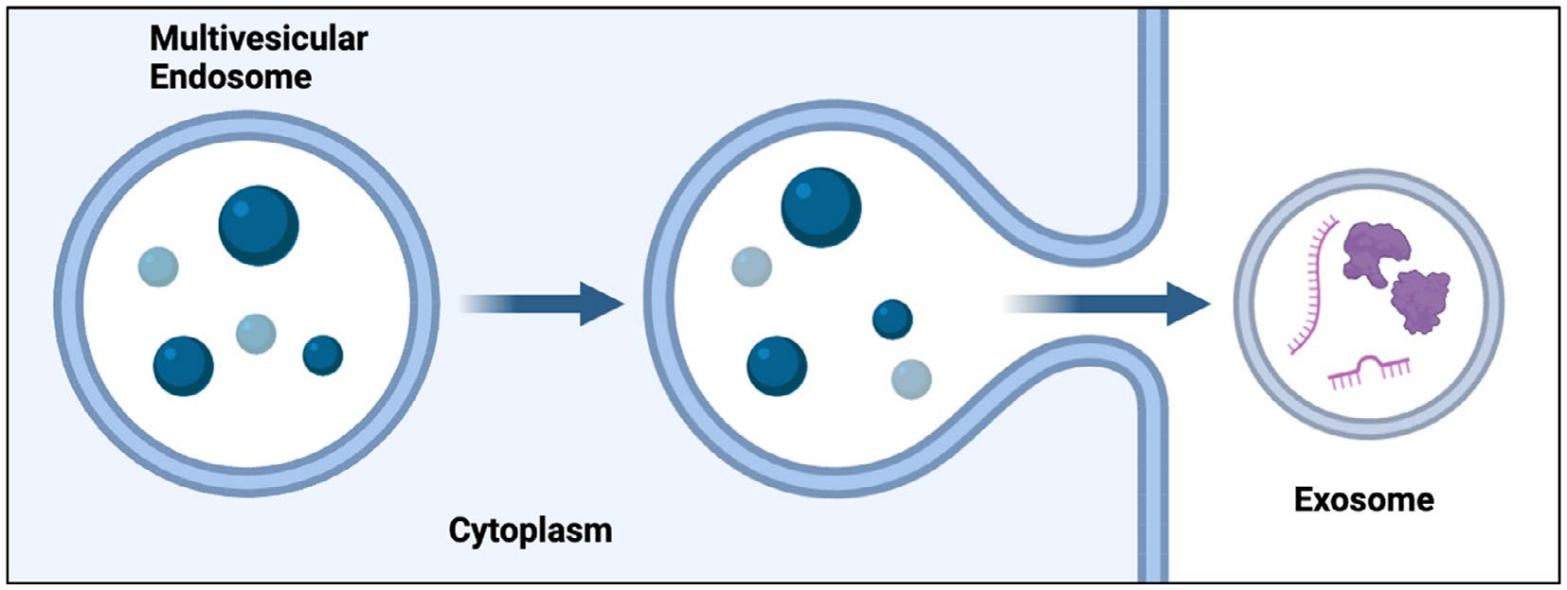
Multivesicular bodies near the plasma membrane are expelled through exocytosis, turning endosomes into exosomes. These exosomes continue various cellular components including protein, transcription factors, RNA, and DNA.

Exosomes have a lipid bilayer membrane that encapsulates a diverse array of biomolecules, including proteins, lipids, and nucleic acids. The lipid bilayer provides structural integrity and aids in the selective packaging and transfer of these molecules [5]. The surface of exosomes is rich in tetraspanins (e.g., CD9, CD63, and CD81), integrins, and major histocompatibility complex (MHC) molecules, which are involved in targeting and adhesion to recipient cells. Internally, exosomes also carry a cargo of cytosolic proteins, enzymes, transcription factors, extracellular matrix proteins, receptors, and nucleic acids such as mRNA, miRNA, and DNA [6] (Figure 1).

The biogenesis of exosomes is a highly regulated process involving several key proteins, particularly members of the Rab family of small GTPases. Rab27, for instance, controls exosome formation at both endosomal and plasma membranes by modulating plasma membrane phosphoinositide dynamics. Another critical player, Rab35, primarily localizes to the plasma membrane and significantly influences exosome release, with its inhibition resulting in a marked reduction in exosome secretion by approximately 50% [7, 8]. Rab11 is implicated in calcium‐induced homotypic fusion and maturation of multivesicular bodies (MVBs), upstream events critical to exosome release [8].

The Ral/Arf6/PLD2/syntenin/Alix axis is also vital for exosome biogenesis. Inhibition of Ral GTPases causes the accumulation of MVBs near the plasma membrane and a substantial decrease in the secretion of exosomes and their marker proteins. This pathway involves Arf6 and PLD2, which are essential for the exosomal release of syndecans (SDCs) and depends on the interaction between syntenin and Alix [9]. These interactions ensure the selective packaging and release of exosomal cargo, vital for their functional roles in recipient cells.

The Endosomal Sorting Complex Required for Transport (ESCRT) machinery, although initially hypothesized to be critical for exosome biogenesis, has shown mixed empirical support. ESCRTs contribute to membrane deformation and scission, processes essential for the formation of intraluminal vesicles within MVBs [10]. However, the necessity of certain ESCRT components, such as the vacuolar protein sorting gene 4 (VPS4) ATPase, appears to be nonessential for the exosomal secretion of common markers like CD63 [8]. This suggests alternative or complementary pathways might be involved in exosome biogenesis which require further elucidation.

### 1.2 Exosomes as Biomarkers

Exosomes may serve as unique biomarkers due to their unique ability to house a diverse array of molecular cargo while protecting those molecules from degradation. These nanometer‐sized vesicles are typically released into various body fluids such as blood, urine, saliva, and cerebrospinal fluid [11]. Their molecular composition closely mirrors the physiological or pathological state of the originating cells. This feature has driven extensive research into their potential use as noninvasive diagnostic and prognostic tools in a range of medical fields, especially oncology, neurodegenerative diseases, and cardiovascular diseases [11].

In the context of cutaneous pigmentation, human keratinocytes release exosomes that modulate pigmentation by transferring specific miRNAs to melanocytes, altering gene expression and tyrosinase activity [12, 13]. These exosomes can either inhibit or promote melanogenesis, as demonstrated by studies showing decreased microphthalmia‐associated transcription factor levels leading to reduced melanin production or increased tyrosinase activity following UVB stimulation. This suggests that keratinocyte exosomes may play a role in detecting hypo‐ and hyperpigmentation disorders through both microphthalmia‐associated transcription factor‐dependent and independent pathways [13].

In melanoma, exosomes from malignant cells can impart metastatic characteristics to benign cells, significantly influencing disease progression [14]. Melanoma‐derived exosomes facilitate communication between primary tumor cells and the microenvironment, promoting vascular leakage, immune cell recruitment, and premetastatic niche formation. The detection of exosomal miRNAs in the plasma of metastatic melanoma patients has shown promise as biomarkers for predicting disease progression [15]. Additionally, increased concentrations of melanoma inhibitory activity, S100B, and specific miRNAs within exosomes highlight their potential as screening tools in dermatology [13].

Merkel cell carcinoma (MCC) is characterized by its aggressive nature and poor prognosis. Proteomics investigations have identified numerous proteins within MCC cell‐derived exosomes that are implicated in motility, metastasis, and tumor progression [16]. These findings lay the foundation for developing liquid biopsy techniques to identify prognostic and diagnostic biomarkers related to MCC progression [13].

Psoriasis, mediated by immune system dysfunction, may also be modulated by exosomes. Exosomes containing phospholipase A2 from mast cells have been implicated in the generation of neolipid antigens recognized by CD1a‐reactive T cells, leading to the production of propsoriatic cytokines IL‐22 and IL‐17a [17]. Increased sensitivity of T cells to phospholipase A2 in psoriatic patients suggests that detecting levels of phospholipase A2 could potentially be a target of interest as a surrogate marker for disease activity. Additionally, targeting exosomal phospholipase A2 and/or CD1a could represent novel therapeutic strategies for managing psoriasis [13].

Exosomes have emerged as potential treatment options in medical aesthetics, contributing to wound repair, antiaging, hyperpigmentation reduction, and hair loss management. These vesicles leverage encapsulated miRNAs and bioactive molecules to orchestrate various biological processes. Mesenchymal stem cell‐derived exosomes (MSCs‐EXOs) are instrumental in regulating macrophage polarization, crucial for modulating inflammatory responses and enhancing wound healing [18]. They facilitate wound closure by promoting hemostasis, suppressing inflammation, accelerating tissue remodeling, and reducing scar formation via signaling pathways such as MAPK and ERK. In antiaging interventions, exosomes derived from human‐induced pluripotent stem cells (iPSC‐EXOs) mitigate cellular senescence by inhibiting markers like β‐galactosidase, thereby preserving cellular functionality. Additionally, exosomes enriched with nicotinamide adenine dinucleotide (NAD+) precursors replenish cellular NAD+ levels, essential for metabolic processes and longevity [19]. In dermatology, exosomes regulate skin pigmentation by modulating melanogenesis pathways, suppressing melanin synthesis through miRNA‐mediated degradation, and influencing key melanogenic proteins like melanocyte‐inducing transcription factor (MITF) [20]. Exosomes from human umbilical cord mesenchymal stem cells (hUCMSCs‐Exos) activate ERK signaling pathways, effectively reducing melanin production and addressing hyperpigmentation. Moreover, in hair loss treatments, exosomes stimulate dermal papilla cell proliferation and activation, crucial for hair growth and maintenance [19]. The modulation of hair follicle signaling and inhibition of suppressive proteins offer novel therapeutic approaches for hair regeneration.

## 2 Methods of Exosome Application

Exosomes are garnering significant attention in medical aesthetics, primarily through topical application and local injection methods. Topical application involves direct skin application or using microneedles and transdermal drug delivery systems to enhance exosome penetration into the skin while local injections refer to subcutaneous injections. Despite the utilization of these approaches, there is no standardized protocol or determination of the most effective administration route. The efficacy of exosomes depends on their source and the delivery method. This section examines the current strategies for exosome administration in medical aesthetics and their efficacy [19].

Topical application of exosomes allows for targeted and controlled dosage [19]. A study on exosomes and miRNA‐221‐3p in a mouse skin injury model showed that both could promote wound healing when applied topically. Transdermal drug delivery systems can enhance the efficacy of exosomes by facilitating the direct delivery of active ingredients beneath the epidermis, significantly improving skin absorption and reducing the loss of active substances [21]. A notable advancement in this domain is the microneedle‐based delivery system, which has shown greater efficacy compared to traditional topical application and subcutaneous injection [22]. For instance, microneedle technology combined with small molecule medication and mesenchymal stem cell (MSC)‐derived exosomes has been shown to enhance treatment outcomes, such as promoting hair regrowth and pigmentation [19, 22].

Local injection of exosomes, particularly subcutaneous injections, offers several advantages, including minimal adverse reactions and ease of administration [10]. Studies have shown that subcutaneous injection of exosomes can expedite wound healing by promoting re‐epithelialization, collagen deposition, and skin cell proliferation. For example, the subcutaneous injection of DF‐Exo significantly improved the healing of diabetic skin lesions in mice [23]. Additionally, adipose‐derived MSC exosomes have been found to decrease transepidermal water loss, enhance stratum corneum hydration, and reduce the expression of inflammatory cytokines, thereby aiding in wound healing. Exosomes incorporated into hydrogels, such as chitosan/silk hydrogels, have demonstrated enhanced wound healing capabilities, suggesting that these combined approaches may offer more targeted and effective treatment options [24].

Despite the promising results of both topical and injectable exosome therapies, there is still a lack of consensus on the most effective method of administration. The choice between these methods may depend on the specific clinical scenario and the source of the exosomes. Transdermal drug delivery, however, is emerging as a preferred method due to its ability to penetrate the stratum corneum, improve the skin's absorption rate of active substances, and minimize side effects [10]. This method holds substantial potential for future applications in medical aesthetics, offering a safer and more convenient alternative to injections.

The effective application of exosomes in medical aesthetics relies heavily on their large‐scale production and preservation. The heterogeneity of exosomes poses challenges to their engineering production. Different methods of separation, purification, and preservation can significantly impact the yield and activity of exosomes [25]. Recent advancements in engineered production technologies, such as the use of polyethylene glycol (PEG) hydrogels and three‐dimensional culture systems, have allowed for the production of stable exosomes with maintained functionality [19].

Limitations of our understanding of exosomes include a lack of standardization of research methods, an incomplete description of their mechanisms of action and the need for further validation of their clinical applications. Furthermore, the development of good manufacturing practice protocols and rigorous quality control measures are necessary to ensure the safe and effective production of exosomes for clinical use [26]. Continued research and innovation in this field will be critical to overcoming these challenges and unlocking the full potential of exosomes in medical aesthetics.

## 3 Direct Therapeutic Application

### 3.1 Skin Rejuvenation

Exosomes have emerged as pivotal regulators in anti‐aging skin rejuvenation, garnering substantial attention due to their capacity for intercellular communication and modulation of human dermal fibroblast (HDF) characteristics. Notably, exosomes derived from three‐dimensional cultured HDF spheroids (3D‐HDF‐exos) have demonstrated significant biological effects, including increased procollagen type I synthesis and reduced MMP‐1 expression via TNF‐α downregulation and TGF‐β upregulation [27]. In both in vitro settings and a nude mouse model of photoaging, 3D‐HDF‐exos have exhibited superior dermal collagen deposition compared to exosomes from bone marrow‐derived mesenchymal stem cells (BMSCs). This suggests their potential to enhance collagen biosynthesis and mitigate inflammation, imparting antiaging properties to dermal fibroblasts [28].

Moreover, exosomes derived from pluripotent stem cells (PSCs), particularly human induced pluripotent stem cell‐derived exosomes (iPSC‐exos), have shown efficacy in ameliorating UVB‐induced photoaging and natural senescence in HDFs [29]. Mechanistically, iPSC‐exos have been found to decrease senescence‐associated β‐galactosidase (SA‐β‐gal) activity and MMP1/3 expression while restoring collagen Type I levels in senescent HDFs [30]. These rejuvenating effects are mediated through the modulation of the TGF‐β receptor 2 pathway by exosomal mmu‐miR‐291a‐3p, derived from embryonic stem cells (ESCs). These findings underscore the therapeutic potential of ESC‐derived exosomes as promising candidates for cell‐free interventions against skin aging, warranting further exploration in clinical applications [28].

### 3.2 Hair Loss

The pathophysiology of hair loss involves complex interactions influenced by genetic predisposition, hormonal fluctuations, immune dysregulation, nutritional deficiencies, pharmaceutical agents, psychological stress, and natural aging processes [31]. Among the key players in HF homeostasis, dermal papilla cells (DPCs) located at the base of the follicle orchestrate hair cycling and regeneration [32]. Exosomes derived from different cellular sources, such as adipose‐derived stem cells (ADSCs) and mesenchymal stem cells (MSCs), have shown promising roles in hair loss therapy through intricate mechanisms involving microRNAs (miRNAs). These miRNAs modulate critical signaling pathways including Wnt/β‐catenin and BMP pathways, crucial for regulating DPC activity and hair follicle stem cell proliferation. Experimental studies indicate that exosome‐mediated delivery of miRNAs promotes the transition of hair follicles from dormant (telogen) to growth (anagen) phases, facilitating hair regeneration, and countering hair loss conditions exacerbated by immune‐mediated factors [28, 33].

Recent advancements underscore the therapeutic potential of exosomes as a cell‐free treatment strategy for immune‐mediated hair loss. Studies have demonstrated that exosomes, particularly those enriched with specific miRNAs, stimulate dermal papilla cell proliferation, enhance hair follicle regeneration, and modulate hair‐inducing signaling pathways [34]. Exosomes from ADSCs have been shown to regulate miR‐22 and the Wnt/β‐catenin pathway, thereby promoting DPC proliferation and maintaining hair follicle viability [35]. Similarly, exosomes derived from human mesenchymal stem cells activate hair‐inducible properties in DPCs by modulating Akt phosphorylation and Bcl‐2 expression, crucial for initiating and sustaining hair growth phases. Despite these promising findings, the clinical translation of exosome‐based therapies for hair loss remains in its infancy, necessitating rigorous clinical trials to validate their efficacy, safety profiles, and long‐term benefits. Addressing these gaps will be critical in harnessing exosomes' full therapeutic potential in combating diverse forms of hair loss and advancing personalized dermatological care strategies [33].

### 3.3 Wound Repair

Exosomes derived from mesenchymal stem cells (MSCs) and induced pluripotent stem cells (iPSCs) exhibit significant potential in wound healing applications due to their ability to facilitate various regenerative processes [36]. Specifically, exosomes from iPSC‐derived MSCs (iPSC‐MSC‐exos) have been shown to accelerate cutaneous wound healing by promoting collagen synthesis, enhancing angiogenesis, and increasing cellular proliferation [37]. These exosomes improve skin cell proliferation through the stimulation of ERK1/2 phosphorylation, ultimately contributing to faster re‐epithelialization, reduced scar formation, and better overall wound outcomes [38]. Moreover, the absence of immunogenicity in iPSC‐MSC‐exos makes them an attractive option for therapeutic use in diverse patient populations [33].

In addition to iPSC‐MSC‐exos, exosomes from iPSC‐derived keratinocytes (iPSC‐KC‐exos) also play a crucial role in wound healing, particularly in enhancing keratinocyte and endothelial cell migration. Studies have demonstrated that iPSC‐KC‐exos promote wound healing in models of deep second‐degree burns through mechanisms involving miR‐762‐mediated regulation of cell migration [39]. These exosomes contribute to re‐epithelialization and angiogenesis, critical phases in the wound healing process. Furthermore, exosomes from human embryonic stem cells (hESC‐exos) have been found to rejuvenate senescent endothelial cells and promote vascular regeneration by activating Nrf2 pathways [40]. Collectively, these findings highlight the promising therapeutic applications of exosomes in wound healing, offering innovative, cell‐free approaches to enhance tissue regeneration and repair [33].

## 4 Limitations

Limitations of exosomes include the difficulty in standardizing exosome production and isolation, as yield and composition can vary significantly depending on source cell type, culture conditions, and isolation techniques. This variability poses challenges in ensuring batch‐to‐batch consistency, which is crucial for clinical application and comparison. Additionally, the lack of standardized protocols for exosome characterization and quantification further complicates the reproducibility and reliability of exosome‐based therapies. Another major challenge is the potential for off‐target effects and unintended biological responses. Although exosomes are generally considered less immunogenic than whole cells, they can still elicit immune responses, particularly when derived from allogeneic sources [41]. Moreover, exosomes carry multiple bioactive molecules that may have unpredictable effects on recipient cells and tissues, raising safety concerns regarding unwanted proinflammatory or oncogenic activities. Furthermore, the scalability of exosome production still requires further development [33]. Addressing these standardization and safety issues is essential to advance the clinical use of exosomes and realize their full therapeutic potential in regenerative medicine and other fields.

## 5 Conclusion

The exploration of exosomes has significantly transformed our understanding of cellular communication and its implications in various physiological and pathological contexts. Originally perceived as mere cellular waste products, exosomes are now recognized as crucial mediators of intercellular communication, involved in a myriad of biological processes and disease mechanisms. This literature review has detailed the multifaceted roles of exosomes, highlighting their complex biogenesis, diverse composition, and substantial functional impact.

Exosomes, with their lipid bilayer membrane and rich cargo of proteins, lipids, and nucleic acids, have demonstrated remarkable potential as biomarkers for various diseases. Their presence in body fluids such as blood, urine, saliva, and cerebrospinal fluid, coupled with their stability and molecular diversity, make them ideal candidates for noninvasive diagnostics. This potential is particularly evident in dermatological conditions. The ability of exosomes to mirror the physiological or pathological state of their cells of origin provides a unique avenue for early diagnosis, monitoring disease progression, and tailoring therapeutic interventions.

In dermatology, exosomes have emerged as pivotal agents in both the diagnosis and treatment of skin‐related disorders. Their role in modulating pigmentation, influencing melanoma progression, and contributing to conditions like Merkel cell carcinoma and psoriasis underscores their diagnostic and therapeutic potential. Additionally, in medical aesthetics, exosomes offer innovative solutions for anti‐aging, wound healing, and hair loss treatments, leveraging their bioactive molecules to enhance cellular functions and tissue regeneration.

Limitations of exosomes include the lack of standardization in isolation and characterization methods, an incomplete understanding of the mechanisms of action, and the need for further safety and efficacy data on the clinical application of exosomes. Continued interdisciplinary research and technological advancements are essential for further applications of exosomes in both clinical and aesthetic dermatology.

In conclusion, the study of exosomes represents a frontier in biomedical science, offering profound insights into cellular communication and promising new directions for disease diagnosis and therapy. As our understanding deepens, the clinical and therapeutic applications of exosomes will likely expand, providing innovative solutions for a range of medical and aesthetic challenges.

## Conflicts of Interest

Victoria Dukharan, Milaan Shah, Luke Broughton, Carol Stegura, Luna Samman, and Nina Schur have no conflicts of interest. Todd Schlesinger serves as a consultant, investigator, speaker, and/or advisor for Abbvie, Almirall, Allergan (An Abbvie company), ASLAN Pharma, Arcutis, Biofrontera, Beirsdorf, Benev, Bristol‐Myers Squibb, Castle Biosciences, Galderma, Eli Lilly, ExoCoBio, Incyte, Janssen, LEO, L'Oreal, Novartis, Pfizer, Regeneron, Sanofi, Sun Pharma, Takeda, UCB Pharma, and Verrica. N.S., L.S., M.S., V.D., L.B., C.S., and T.S. All contributed to the planning, review, synthesis, and writing of the manuscript. All authors have read and approved the final manuscript.

## Data Availability

Data sharing not applicable to this article as no datasets were generated or analysed during the current study.
